# InfectA-Chat, an Arabic Large Language Model for Infectious Diseases: Comparative Analysis

**DOI:** 10.2196/63881

**Published:** 2025-02-10

**Authors:** Yesim Selcuk, Eunhui Kim, Insung Ahn

**Affiliations:** 1 Department of Applied AI KISTI School University of Science and Technology Daejeon Republic of Korea; 2 AI Data Research Team, Department of Data-Centric Problem Solving Research Division of S&T Digital Convergence Korea Institute of Science and Technology Information Daejeon Republic of Korea; 3 Department of Data-Centric Problem Solving Research Korea Institute of Science and Technology Information Daejeon Republic of Korea

**Keywords:** large language model, Arabic large language models, AceGPT, multilingual large language model, infectious disease monitoring, public health

## Abstract

**Background:**

Infectious diseases have consistently been a significant concern in public health, requiring proactive measures to safeguard societal well-being. In this regard, regular monitoring activities play a crucial role in mitigating the adverse effects of diseases on society. To monitor disease trends, various organizations, such as the World Health Organization (WHO) and the European Centre for Disease Prevention and Control (ECDC), collect diverse surveillance data and make them publicly accessible. However, these platforms primarily present surveillance data in English, which creates language barriers for non–English-speaking individuals and global public health efforts to accurately observe disease trends. This challenge is particularly noticeable in regions such as the Middle East, where specific infectious diseases, such as Middle East respiratory syndrome coronavirus (MERS-CoV), have seen a dramatic increase. For such regions, it is essential to develop tools that can overcome language barriers and reach more individuals to alleviate the negative impacts of these diseases.

**Objective:**

This study aims to address these issues; therefore, we propose InfectA-Chat, a cutting-edge large language model (LLM) specifically designed for the Arabic language but also incorporating English for question and answer (Q&A) tasks. InfectA-Chat leverages its deep understanding of the language to provide users with information on the latest trends in infectious diseases based on their queries.

**Methods:**

This comprehensive study was achieved by instruction tuning the AceGPT-7B and AceGPT-7B-Chat models on a Q&A task, using a dataset of 55,400 Arabic and English domain–specific instruction–following data. The performance of these fine-tuned models was evaluated using 2770 domain-specific Arabic and English instruction–following data, using the GPT-4 evaluation method. A comparative analysis was then performed against Arabic LLMs and state-of-the-art models, including AceGPT-13B-Chat, Jais-13B-Chat, Gemini, GPT-3.5, and GPT-4. Furthermore, to ensure the model had access to the latest information on infectious diseases by regularly updating the data without additional fine-tuning, we used the retrieval-augmented generation (RAG) method.

**Results:**

InfectA-Chat demonstrated good performance in answering questions about infectious diseases by the GPT-4 evaluation method. Our comparative analysis revealed that it outperforms the AceGPT-7B-Chat and InfectA-Chat (based on AceGPT-7B) models by a margin of 43.52%. It also surpassed other Arabic LLMs such as AceGPT-13B-Chat and Jais-13B-Chat by 48.61%. Among the state-of-the-art models, InfectA-Chat achieved a leading performance of 23.78%, competing closely with the GPT-4 model. Furthermore, the RAG method in InfectA-Chat significantly improved document retrieval accuracy. Notably, RAG retrieved more accurate documents based on queries when the top-k parameter value was increased.

**Conclusions:**

Our findings highlight the shortcomings of general Arabic LLMs in providing up-to-date information about infectious diseases. With this study, we aim to empower individuals and public health efforts by offering a bilingual Q&A system for infectious disease monitoring.

## Introduction

### Motivation

Throughout human history, there has been a continuing battle against infectious diseases. Bacteria, parasites, and viruses can spread quickly among people, resulting in a wide range of illnesses and significantly affecting public health. Despite major advancements in medicine and public health, new infectious diseases continue to arise. The recent COVID-19 outbreak, caused by the SARS-CoV-2 virus, is an example of the continuing existence and profound impacts of infectious diseases all over the world. Beyond COVID-19, there has been an increase in other infectious diseases around the world. For instance, the Middle East respiratory syndrome coronavirus (MERS-CoV), a zoonotic infection primarily occurring in the Arabian Peninsula, poses a significant public health threat. Globally, as of April 29, 2024, there have been 2622 reported cases of MERS-CoV, including 950 deaths [1]. Similarly, cholera, a bacterial infection causing severe dehydration in the human body, has been reported, particularly in Africa and Asia. The World Health Organization (WHO) reported 787,813 cases and 5586 deaths across 31 countries as of March 14, 2024 [2]. In addition, other infectious diseases, such as dengue fever and avian influenza, have become a challenge to public health around the world. These examples indicate that society needs effective preventive measures to eliminate the adverse effects of infectious diseases.

At this point, identifying the patterns of disease spread is quite important for coping with infectious diseases. By identifying the significant factors for disease persistence, several strategies can be developed for public health prevention and control. Regular monitoring operations play a critical role, allowing us to track disease outbreaks and take preventive measures in advance. Numerous platforms collect and make large amounts of surveillance data publicly accessible for this purpose. The WHO stands as a key leader in the public health domain, delivering up-to-date outbreak information as well as regional data and statistics.

Similarly, the European Centre for Disease Prevention and Control (ECDC) is dedicated to disease surveillance, outbreak preparedness, scientific advice, and public health training [3]. These platforms offer real-time disease monitoring for individuals and public health efforts, fostering rapid responses to outbreaks. While these platforms provide a large amount of information, they bring challenges for individuals seeking specific information. By delivering information primarily through long news articles or reports, they offer timely updates about infectious diseases. In this context, large language models (LLMs), such as GPT-4 [4], Falcon [5], Pathways Language Model (PaLM) [6], and LLaMA-2 [7] have recently played a significant role in providing information about various fields, including the medical and public health domains, in multiple languages. These models, with their ability to understand and generate humanlike text in multiple languages, have become popular tools for public use.

### The Rise of LLMs in the Medical Domain

Deep learning has yielded state-of-the-art results in various fields, such as computer vision, natural language processing (NLP), public health, biology, and recommendation [8]. Among these advancements, the field of LLMs has rapidly evolved in various ways. An LLM is a deep learning–based artificial neural network, distinguished from traditional machine learning models by its training on vast amounts of textual data [9]. Specifically, following the introduction of the transformer architecture, researchers began to explore pretrained, large-scale, transformer-based models trained on vast amounts of data [10]. Models such as the GPT series and Bidirectional Encoder Representations from Transformers (BERT) [11] demonstrated the effectiveness of pretraining transformer models on large corpora. Particularly, the GPT family has attracted significant attention. Further enhancements in the GPT model led to more sophisticated LLMs, such as GPT-4 [4], which demonstrated outstanding performance in natural language understanding, generation, and various NLP tasks. These models have opened new research directions toward artificial general intelligence [12].

While models like the GPT family have brought greater capabilities, they pose challenges due to being closed-source models. To address this issue, the research community in the NLP field has shifted its focus to open-source alternatives. At this point, LLaMA [13], LLaMA-2 [7], and Alpaca are important examples of such endeavors [12]. In February 2023, the first LLaMA models [13], which include parameters ranging from 7B to 65B, were released. Trillions of tokens from publicly accessible datasets were used to pretrain these models. The LLaMA architecture includes the SwiGLU activation function and rotary positional embeddings, which are enhancements over the GPT decoder-only architecture, which brings significant performance with LLaMA-13B outperforming GPT-3 (175B) on most benchmark datasets [13]. This release was significant because it inspired the development of other open-source models, such as Mistral [14].

Despite the advancements in multilingual LLMs, such as GPT and LLaMA, they are still primarily trained on English datasets, limiting their ability to extend comprehension and generation capabilities to languages other than English. This situation highlights the necessity for non-English LLMs, which are pretrained predominantly in languages other than English, such as Arabic. Arabic is one of the world’s most spoken languages, with >400 million speakers, but it is underrepresented in the LLM field so far. To address this issue, several Arabic LLMs have been developed with various architectures. AraBERT [15], QARiB [16], JABER and SABER [17], CAMeLBERT [18], and AraELECTRA [19] are examples of encoder-only models, while ARABART [20], ARAGPT2 [21], Jais [22], and ALLaM [23] are categorized as decoder-only models [22]. Recently, the AceGPT model has gained prominence for its emphasis on cultural sensitivity and the incorporation of local values, setting it apart from other Arabic LLMs. Comprehensive evaluations show that AceGPT demonstrates state-of-the-art performance among open Arabic LLMs across various benchmarks [24].

Building upon the success of general LLMs, researchers explored adapting these pretrained models to the specific needs of the medical domain. This involved leveraging large-scale biomedical corpora like PubMed Central articles. For instance, PubMedBERT [25] was pretrained on PubMed data, while ClinicalBERT [26] was pretrained on the Medical Information Mart for Intensive Care III (MIMIC-III) dataset. In addition, BERT for Biomedical Text Mining (BioBERT) [27] was pretrained on PubMed and PubMed Central Dataset datasets [28]. However, the rise of big autoregressive generative models, such as GPT and LLaMA, led to the use of decoder-only architectures to train medical LLMs with medical domain data. GatorTronGPT [29], a model that is similar to GPT-3, was pretrained on 227 billion words of mixed clinical and general English data, boasting 20B parameters. In terms of medical reasoning, Flan-PaLM [30] and PaLM-2 [31] are examples of pretrained models, both with 540B parameter sizes. MEDITRON [32] is one of the fine-tuned models that shows competitive performance with the PaLM-2 and Flan-PaLM models [32]. These pretrained language models can be fine-tuned for many downstream tasks, leading to the development of several specialized medical LLMs such as ChatDoctor [33], MedAlpaca [34], PMC-LlaMA [35], BenTsao [36], and Clinical Camel [37], in addition to publicly available general-purpose LLMs [28]. While the number of medical LLMs is growing, only a few Arabic language models were developed for medical domains, such as Bilingual Medical Mixture of Experts Large Language Model (BiMediX) [38] and Apollo [39]. Despite their abilities to assist society and medical efforts, there are still limitations, particularly for infectious disease monitoring, due to being trained on limited infectious disease surveillance data.

### The Contributions of InfectA-Chat

To tackle this issue, we present InfectA-Chat, a cutting-edge Arabic LLM designed to improve AceGPT’s abilities to understand and generate text in Arabic in the domain of infectious diseases by providing information on the latest outbreaks of infectious diseases based on user queries. We prepared and curated an instruction-following dataset for the question and answer (Q&A) task based on infectious disease–related news articles, using instruction tuning on the AceGPT-7B and AceGPT-7B-Chat models. To facilitate efficient fine-tuning of these models, we adopted the low-rank adaptation (LoRA) [40] method, which is particularly beneficial in scenarios with limited computational resources or data availability. Unlike other parameter-efficient fine-tuning (PEFT) approaches, such as adapters or prompt tuning, LoRA integrates trainable rank decomposition matrices within specific layers of the pretrained model. This enables the model to capture the necessary low-rank updates for fine-tuning without significantly increasing inference latency. LoRA’s efficiency makes it a tempting solution for real-world applications that need to preserve performance while decreasing computational expenses. Therefore, we use LoRA as a PEFT method for these reasons [12]. Our model addresses the need for an interactive and real-time infectious disease tracking system with potential applicability across different languages and public health domains. To sum up, the contributions made by the InfectA-Chat model are mentioned subsequently.

First, we enhanced AceGPT’s capabilities in the infectious diseases domain by extending its dataset with related articles. This improved model, named InfectA-Chat, is an Arabic and English Q&A application dedicated to infectious disease tracking. It offers valuable resources to the NLP and public health communities and empowers individuals to stay informed about infectious disease trends and developments.

Second, we leveraged LoRA to facilitate PEFT, optimizing performance despite constrained computational resources.

Third, we used the retrieval-augmented generation (RAG) pipeline to demonstrate that reapplying instruction tuning is unnecessary when updating the model with recent data. To validate this approach, we assessed the performance of the RAG model using the latest infectious disease–related information on InfectA-Chat and compared its efficiency to that of state-of-the-art models.

Fourth, we encourage additional research and collaboration within the NLP and public health communities by making our study publicly available [41].

## Methods

### Overview

InfectA-Chat is based on the AceGPT-7B-Chat model, a fine-tuned version of the AceGPT model that was derived from the LLaMA2-7B model by further training using a large corpus of Arabic text, containing 19.2B Arabic and 10.8B tokens in English. The AceGPT methodology addresses the localization concerns in the Arabic LLMs that contain unique, cultural characteristics inappropriately. To address this issue, it provides localized pretraining, localized supervised fine-tuning, and reinforcement learning from artificial intelligence feedback with localized preference data. During the localized pretraining, to adapt the English-focused LLaMA2 model to Arabic, the researchers leveraged a variety of Arabic datasets, including Arabic text 2022, Arabic Wikipedia, CC100, and OSCAR3. To prevent the loss of knowledge in English text, they obtained a dataset from SlimPajama. In addition, the original vocabulary of LLaMA2, which contains 53 Arabic letters, is retained to reduce training costs. Furthermore, AceGPT was fine-tuned with localized instructions and responses to allow the model to follow Arabic user instructions, using questions derived from real-world scenarios. In the evaluation results, it is shown that AceGPT becomes a state-of-the-art model among open Arabic LLMs across various benchmarks [24].

**Figure 1:**
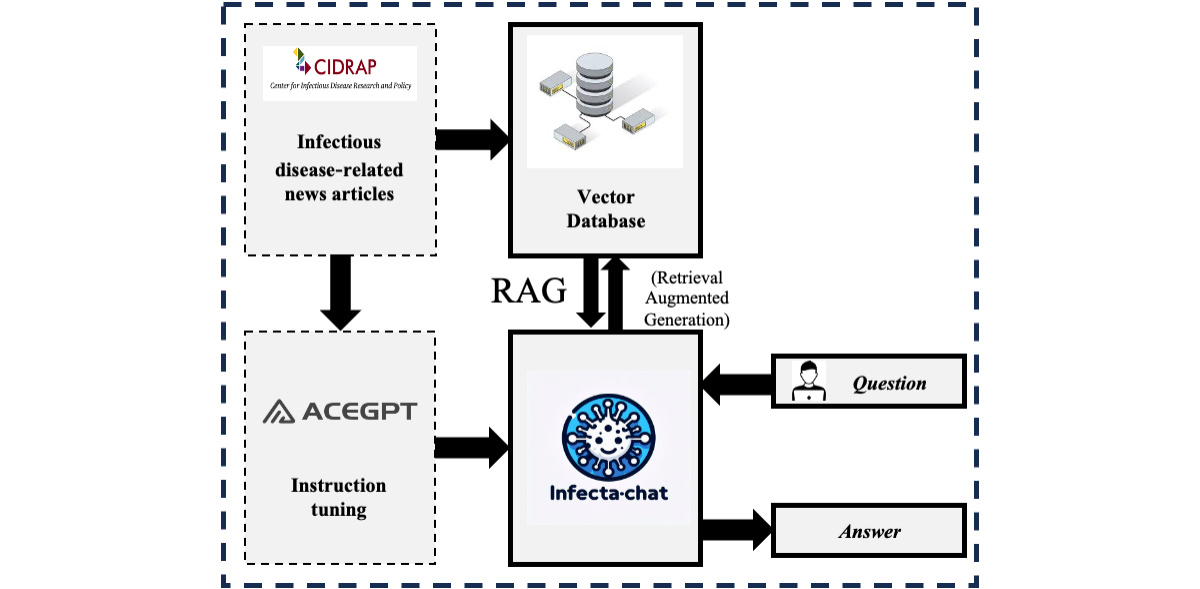
Modeling of InfectA-Chat. RAG: retrieval-augmented generation.

Due to AceGPT’s advanced capabilities among Arabic LLMs, the development of InfectA-Chat followed a 2-stage pipeline based on the AceGPT structure (Figure 1). First, we used instruction tuning on the AceGPT-7B and AceGPT-7B-Chat models [24] using the instruction-following dataset. The LoRA method was chosen for this stage, enabling PEFT to minimize computational cost while maintaining performance. This resulted in the creation of the InfectA-Chat (based on AceGPT-7B) and InfectA-Chat (based on AceGPT-7B-Chat) models. Following the instruction tuning, we conducted a comprehensive GPT-4 evaluation [42] to compare the performance of InfectA-Chat models (based on AceGPT-7B and AceGPT-7B-Chat) against each other, state-of-the-art models, and Arabic LLMs such as GPT-3.5 [43], GPT-4 [4], Gemini [44], AceGPT [24], and Jais [22]. During the GPT-4 evaluation, instruction-tuned models were assessed using English and Arabic real-world data pertaining to infectious diseases, reflecting actual outbreak information and other relevant details extracted from articles from the Center for Infectious Disease Research and Policy (CIDRAP) [45] platform. By prompting the GPT-4 model to compare the answers of comparison models with relevant queries and articles, GPT-4 evaluated the performance of models. To ensure the consistency and trustworthiness of the GPT-4 evaluation method, we also implemented variance analysis. In addition, to evaluate the generalizability of the best-performing InfectA-Chat model, we conducted cross-domain testing on an Arabic benchmark dataset, comparing it with other Arabic LLMs and state-of-the-art models. In the second stage, we leveraged the RAG [46] pipeline based on the superior InfectA-Chat model, which was identified in the GPT-4 evaluation. Applying RAG allowed us to update the model’s data with the latest information in the infectious disease domain without requiring further instruction tuning while maintaining performance. This 2-stage approach empowered InfectA-Chat to effectively address the Q&A task within the infectious disease domain.

### Data Collection and Preprocessing

**Figure 2:**
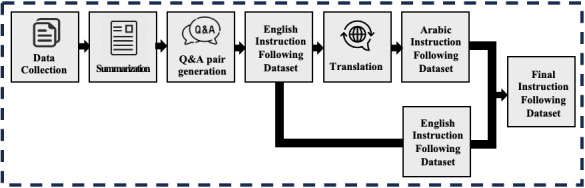
The overall pipeline of data collection and preprocessing. Q&A: question and answer.

Data preprocessing plays a vital role in training high-quality LLMs [12], especially when working with the limited structured data available on infectious diseases in English and Arabic. To address this gap, we conducted many steps to get high-quality Arabic and English instruction–following data. The overall pipeline of the data preprocessing flow is shown in Figure 2. As a first step, we selected CIDRAP [45] as our major data source due to its reputation for publishing timely and well-researched articles on infectious diseases. CIDRAP’s information is both credible and extensive, with daily updates on growing health threats globally, making it a great foundation for our dataset. Given the challenges of sourcing accurate data on infectious diseases, CIDRAP’s meticulous reporting and in-depth coverage were vital for our study. Therefore, we conducted web scraping to gather data from the CIDRAP website, focusing on the MENA (Middle East and North Africa) region, including 20 countries, with published dates between February 20, 2020, and February 20, 2024. This resulted in 2770 English articles collected from CIDRAP’s website. Subsequently, we further enriched the articles by adding essential information, such as the country, title, and publication date of the article. The collected articles had varying lengths, some exceeding the maximum context length of InfectA-Chat, which is 2048. This posed a challenge because long articles could not be processed directly within the model. To tackle this limitation and enhance the dataset’s usability, we implemented 2 summarization methods, GPT-3.5-Turbo [47] and Gensim [48]. We implemented 2 summarization methods as a more cost-effective alternative compared to relying solely on GPT-3.5-Turbo, which incurs application programming interface use costs. By incorporating a dual-approach strategy, we could streamline the summarization process, reducing the application programming interface cost while preserving the essential information in the summarized articles for model training. The first approach was summarization by using Gensim (RARE Technologies Ltd), a popular open-source Python toolkit that is well-suited for NLP tasks, such as text summarization. Gensim provides powerful summary algorithms that effectively condense articles while retaining key information, allowing users to define a desired word count. In this phase, we established a word count that is consistent with the 2048-token limit, allowing us to efficiently summarize our collection of 2770 articles. The second approach used GPT-3.5-Turbo-0125, an autoregressive model known for providing high-quality text generation based on various tasks, including summarization. By prompting the GPT-3.5-Turbo-0125 model to produce summaries based on our specified token count, we were able to distill our collected articles effectively. Using both approaches, the articles were effectively summarized while retaining essential information, expanding their maximum context length to 2048. Given that we used 2 distinct summarization approaches, we are generating 2 types of summarized articles. Therefore, these 2 approaches not only addressed the context length limitation but also effectively augmented the data by leveraging their advanced text summarization capabilities.

This study focused on building an instruction-following dataset based on a Q&A task. To generate Q&A pairs from summarized articles, we used the GPT-3.5-Turbo-0125 model, generating 5 high-quality Q&A pairs per summarized article. During the Q&A generation process, we particularly instructed the model to generate Q&A pairs that are explicit and directly related to the context and to include date information in the questions by avoiding ambiguous phrases, such as “last week,” “over the past week,” and “this year.” Therefore, we built an English instruction–following dataset that contains 27,700 Q&A pairs based on each summarized article as shown in Textbox 1. Textbox 1 presents the example of English and Arabic instruction–following data with their translation. Because there is a lack of Arabic data in the domain of infectious diseases, we needed to translate our English instruction–following data into Arabic. To generate an Arabic instruction–following dataset, we used the GPT-3.5-Turbo-0125 model to translate all the English instruction–following data into Arabic. As a final step, we concatenated the English and Arabic instruction–following datasets, yielding 55,400 Q&A pairs with a token size of 54.5B, based on the AceGPT-7B-Chat tokenizer. We used the original vocabulary of the AceGPT-7B model, which has a size of 32,000 tokens and contains 53 Arabic letters. The 55,400 generated instruction-following data points, which reflect real-world information about infectious diseases, were divided into training, validation, and test datasets in proportions of 90%, 5%, and 5%, respectively.

**Example of English and Arabic instruction–following data.**

**English instruction–following data**

Below is an instruction that describes Q&A task. Write a response to complete the request appropriately.

### Instruction: How many patients were reported with MERSCoV cases in Saudi Arabia between December 29, 2021, and October 31, 2022?

Title: WHO details 4 more MERS cases in Saudi Arabia

Date: November 17, 2022

Country: Saudi Arabia

Source: “https://www.cidrap.umn.edu/mers-cov/who-details-4-more-mers-cases-saudi-arabia”

Contents: The World Health Organization (WHO) yesterday posted an update on Middle East respiratory syndrome coronavirus (MERS-CoV) in Saudi Arabia that covers 4 cases reported by the country since the WHO’s last update in April. Three of the patients had recent contact with camels. No secondary cases were reported, and none were fatal. The WHO said the 4 MERS-CoV cases were reported between December 29, 2021, and October 31, 2022. Symptom onset ranged from December 27 to June. All patients—3 men and a woman, were aged 23 to 68 years and were not health care workers. All had underlying conditions and were hospitalized. The men had contact with camels and had consumed camel milk. The agriculture ministry’s related investigations identified the virus in camels in those 3 instances. So far, the source of virus exposure for the woman aged 23 years is not known. Two patients are from Riyadh province, from the cities Afif and Al Kharj. The others are from Mecca in Mecca province and Buridah in Al Qassim province. The WHO noted that the number of MERS-CoV cases has declined since the start of the COVID-19 pandemic, likely due to health officials prioritizing COVID-19 activities. In addition, the WHO said COVID-19 protective measures may have reduced MERS-CoV transmission. However, the WHO said MERS-CoV circulation continued in dromedary camels and that the zoonotic threat remains. The WHO said the latest cases from Saudi Arabia lift the global total since September 2012 to 2600 cases, 935 of them fatal.

### Response: In February, Saudi Arabia reported 18 MERS-CoV cases, with 5 of them being fatal, from 9 different cities.

**Arabic instruction–following data**

Below is an instruction that describes Q&A task. Write a response to complete the request appropriately.

### Instruction: ما هي الدول التي ذكرت أنها سجلت المزيد من حالات شلل الأطفال، وما هي الدولة التي أبلغت عن أول حالات ناجمة عن اللقاح؟

Title: مسح الأخبار ليوم 25 سبتمبر 2020

Date: 25 سبتمبر 2020

Country: السودان

Source: “https://www.cidrap.umn.edu/salmonella/news-scan-sep-25-2020”

Contents: وقد أثر تفشي السالمونيلا المرتبط بالفطر المجفف في الأطباق المقدمة في مطاعم الرامن على 41 شخصًا في 10 ولايات، ومن المرجح أن يكون المصدر هو فطر الأذن الخشبية الذي توزعه شركة Wismettac Asian Foods. وفي أستراليا، أظهر استخدام المضادات الحيوية انخفاضًا من عام 2013 إلى عام 2017، وإن كان لا يزال أعلى مقارنة بدول مثل هولندا والسويد. وتستمر الجهود لتحسين وصف المضادات الحيوية. بالإضافة إلى ذلك، أبلغت خمس دول عن المزيد من حالات شلل الأطفال، حيث أبلغ جنوب السودان عن أولى حالاته المشتقة من اللقاح.

### Response: وأبلغت خمس دول عن المزيد من حالات شلل الأطفال، حيث أبلغ جنوب السودان عن أول حالاته المرتبطة باللقاح.

English version: Below is an instruction that describes Q&A task. Write a response to complete the request appropriately.

### Instruction: Which countries were mentioned to have reported more polio cases, and which country reported its first vaccine-derived cases?

Title: News Scan for September 25, 2020

Date: September 25, 2020

Country: Sudan

Source: “https://www.cidrap.umn.edu/salmonella/news-scan-sep-25-2020”

Contents: A Salmonella outbreak linked to dried mushrooms in dishes served at ramen restaurants has affected 41 people in 10 states, with the likely source traced to wood ear mushrooms distributed by Wismettac Asian Foods. In Australia, antibiotic use has shown a decline from 201,314 to 201,718, though still higher compared to countries like the Netherlands and Sweden. Efforts to optimize antibiotic prescribing continue. In addition, 5 countries reported more polio cases, with South Sudan reporting its first vaccine-derived cases.

### Response: Five countries reported more polio cases, with South Sudan reporting its first vaccine-linked case

### Supervised Fine-Tuning (Instruction Tuning)

Pretrained LLMs often struggle to effectively track user instructions and may generate inadequate output based on given instructions. This limitation stems from their primary goal, which is predicting the next token rather than generating output directly in response to the given instruction, as described in equation 1; the loss of pretraining *L_PT_* is updated with the next token prediction based on the pretraining dataset *D_PT_* [12].



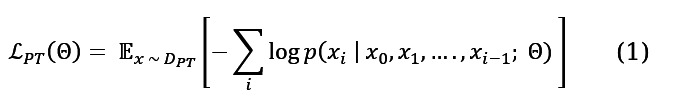



**Figure 3:**
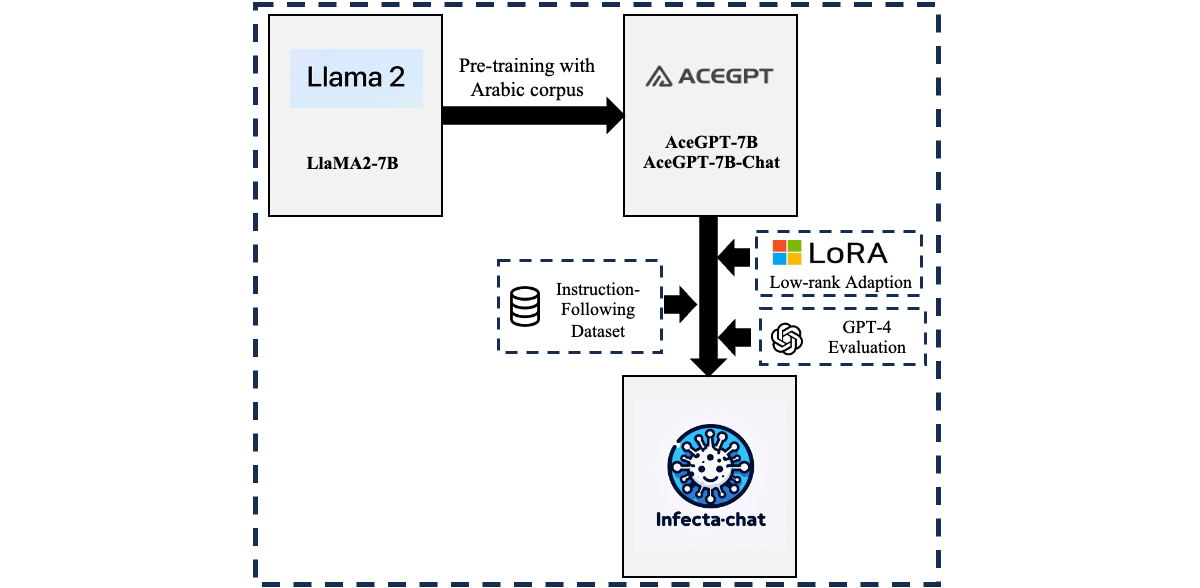
The overall pipeline of supervised fine-tuning (instruction tuning).

To overcome this challenge, the supervised fine-tuning, also known as instruction tuning method, is often used, which allows pretrained LLMs to be adapted to a specific task using instruction-following data. The instruction tuning method consists of a prompting model with instructions, which are encapsulated by a prompt template. The model is then trained by using given instructions and generates the proper output. In this study, we applied instruction tuning on the AceGPT-7B and AceGPT-7B-Chat models using Arabic and English instruction–following data as shown in Figure 3. Our instruction-following dataset follows a format that is similar to the Alpaca dataset [49], where each data point contains an instruction and output. The template for fine-tuning is shown in Textbox 2.

**Prompting template of instruction tuning.**

“Below is an instruction that describes Q&A task. Write a response to complete the request appropriately.

### Instruction: {question}\n {Title: title, Date: date, Country: country, Source: source, Contents: article}

### Response:”

In our instruction-following dataset, the prompt template’s instruction part combines both a question and an article, distinguished by “\n,” which differs from the structure of the Alpaca dataset. Admittedly, we enhanced the dataset by including important details for articles, such as title, publication date, country, and source alongside the main context of the article. This also helped with accurate article retrieval during the application of RAG.

During traditional training, all parameters of LLMs are often updated, which is not cost-effective and takes a lot of time [12]. To tackle this, PEFT methods are generally applied during the process of fine-tuning, such as prefix tuning, adapter tuning, and LoRA, which have been recently studied as representative PEFT methods. Among the 3 representative PEFT models, prefix tuning offers limited performance with no added latency, while adapter tuning prioritizes performance at the cost of increased latency [50]. In addition, prefix tuning is difficult to optimize, as the training process can be unstable due to nonmonotonic changes in training loss [40].

Compared to other methods, LoRA reduces computation by applying, as its name implies, “low-rank adaptation” weights to the query and key operations of the attention module. The feed-forward operation remains the same as in general attention modules, while the backward operation uses low-rank weights to perform calculations for each layer. This entails mapping to a smaller dimension, applying nonlinear activation, and then returning to the original dimension size. LoRA emerges as the most attractive solution for PEFT in LLMs due to its superior mix of performance, efficiency, and latency. Therefore, in this study, we use LoRA during the fine-tuning of the AceGPT-7B and AceGPT-7B-Chat models by integrating LoRA adapters into the weights of the multilayer perceptron layers and attention modules. The application of LoRA to all linear transformer blocks has been confirmed in quantized LoRA [12,51].

During the fine-tuning process, the loss is only calculated on the {output} part of the input sequence and can be expressed as shown in equation 2. In this equation, *L_SFT_* represents loss of instruction tuning. Θ represents the model parameters, while *D_SFT_* is the fine-tuning dataset; *x*=(*x*_0_, *x*_1_, ...) represents the tokenized input sentence [12].



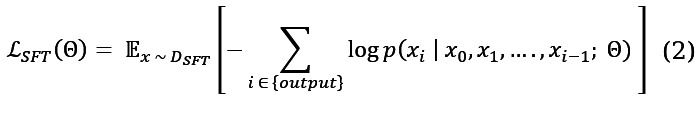



### RAG Based on InfectA-Chat

For building domain-specific LLMs like InfectA-Chat, instruction tuning has emerged as a highly effective method, demonstrating impressive results in tasks such as Q&A. However, instruction tuning LLMs for specific tasks has various limitations that can hinder their effectiveness. Instruction tuning mostly relies on the quality and quantity of training data. In addition, fine-tuned LLMs may struggle to generate factually correct and contextually relevant responses, particularly when the training data are limited, known as “hallucinations.” Especially in domains such as infectious diseases, where up-to-date information is crucial for accurate monitoring, repeatedly applying instruction tuning to new datasets becomes impractical due to time and resource constraints. RAG [46] provides a convincing method that complements instruction tuning while resolving its limitations. RAG uses the power of accessing a wide external knowledge base to generate text for a query. This allows RAG to gather relevant information specific to the prompt, yielding more informative and accurate outputs.

RAG typically consists of 2 main components: a retriever and a generator. The retriever first identifies relevant documents or information from the external knowledge source based on the input prompt. The retrieved information is then passed on to the generator, which is often a fine-tuned LLM. The generator produces the final result by combining both the retrieved information and its own internal knowledge to produce the final output. RAG prioritizes article quality. To enable more accurate retrieval, essential information, such as country, title, source, and published date, was included in the main content of the article.

**Figure 4:**
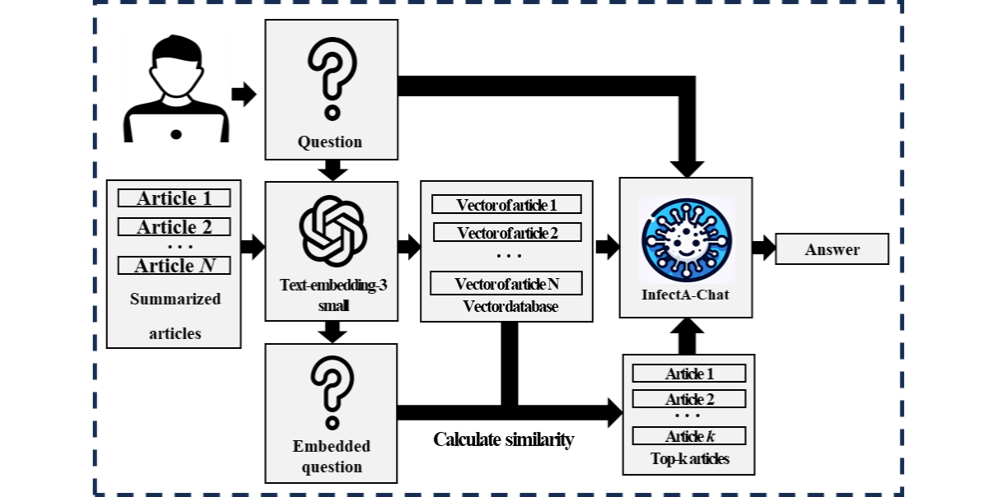
The overall pipeline of Retrieval-Augmented Generation.

The overall pipeline of RAG is depicted in Figure 4. Initially, summarized articles were embedded using text-embedding-3-small model [52]. When a user query was received, it was embedded as a vector by the text-embedding-3-small model, which calculates the similarity between a user query and the vector database of summarized news articles. As a similarity metric, the cosine similarity was used. Following the similarity calculation, the top-k articles were selected and sent to InfectA-Chat along with the query. Top-k refers to the specified number of articles that have a high correlation with the query and are set by the developer during the RAG process. Once the RAG pipeline recalls the highly correlated articles with the query, the model can generate an answer based on the selected top-k articles and the query. By using RAG, the model can provide more factually and contextually accurate answers.

Our goals included not only preventing hallucinations when applying RAG but also avoiding the need for repeated instruction tuning when updating InfectA-Chat’s data. To demonstrate the necessity of the RAG pipeline for updating InfectA-Chat’s data, we prepared 50 instruction-following data points with published dates from March to June 2024, ensuring that they were not part of the original InfectA-Chat training data. During the recent data preparation, we specifically emphasized the importance of including the date in the questions. We applied the RAG pipeline using these recent 50 instruction-following data to InfectA-Chat, GPT-3.5, and GPT-4, using GPT-4 evaluation for a comprehensive comparison.

In addition, we investigated the impact of the top-k parameter on information retrieval within the RAG framework. Specifically, we compared the retrieval performance using top-1, top-3, and top-5 settings in the RAG process.

### Ethical Considerations

This study used publicly available data sourced from the CIDRAP. The preprocessing steps ensured that the data protects the individual’s privacy. Due to these factors, no ethics review was required. All analyses and findings are presented in alignment with the standards set by *JMIR Medical Informatics* and aim to contribute to the field of infectious disease research without compromising ethical norms.

## Results

### Experimental Setup

LLM training represents a significant engineering problem. Due to the enormous number of the model parameters, a large-scale distributed training framework is required, allowing the use of several graphics process units (GPUs) across numerous processing nodes. The InfectA-Chat models were trained with a total of 4 Nvidia A100 80GB GPUs on 2 nodes, provided by the Korea Institute of Science and Technology Information. To leverage complementary parallelism for faster and more memory-efficient training, we used the DeepSpeed framework during instruction tuning. DeepSpeed [53] is a robust open-source library that optimizes deep learning training for *PyTorch* by reducing memory use and increasing training efficiency. It solves the issues of training LLMs on restricted hardware by using approaches, such as Zero Redundancy Optimizer, which distributes model parameters and gradients over numerous processors, significantly reducing memory footprint. In this study, Zero Redundancy Optimizer stage 2 was applied, enabling the distribution of optimizer states and gradients across GPUs, thereby facilitating efficient large-scale training.

Instruction tuning was performed using 49,860 instruction-following data points, containing 49 million tokens, each for 2 models: AceGPT-7B and AceGPT-7B-Chat, while validation and test datasets comprised 2770 instruction-following data points, containing 2.8 and 2.7 million tokens, respectively. All models were trained with a batch size of 12 per device, resulting in a total batch size of 1296. The AdamW optimizer was used, using a cosine learning rate scheduler with a warmup ratio of 0.03% of steps and a peak learning rate of 0.0001. A weight decay of 0.01 was applied along with a maximum sequence length of 2048 as well as a gradient accumulation of 27. In addition, all models included LoRA with a rank of 64, an α of 128, weights of Q, K, V, O, and multilayer perceptron, and a dropout rate of 0.05. The hyperparameters of instruction tuning of both AceGPT-7B and AceGPT-7B-Chat models are listed in detail in Table 1 [12]. Following instruction tuning, a comprehensive comparison analysis was conducted to evaluate each model’s performance. Using GPT-4 evaluation, an advanced model published by OpenAI, renowned for its state-of-the-art performance in text generation among LLMs, provided a robust assessment. By using an evaluation prompt, GPT-4 systematically evaluated the models by comparing their responses to specific questions and articles (Textbox 3). The comparison analysis was performed in 3 distinct phases.

**Table 1:** Hyperparameters.

Base model	AceGPT-7B	AceGPT-7B-Chat
Vocabulary size (tokenizer)	32,000 (AceGPT-7B)	32,000 (AceGPT-7B-Chat)
Training dataset	49,860 (49 million tokens)	49,860 (49 million tokens)
Validation dataset	2770 (2.8 million tokens)	2770 (2.8 million tokens)
Test dataset	2770 (2.7 million tokens)	2770 (2.7 million tokens)
GPU^a^	Nvidia A100×4	Nvidia A100×4
Batch size per device	12	12
Training time per 1 step	846.42 s	846.42 s
Gradient accumulation	27	27
Total batch size	48	48
DeepSpeed	Zero stage 2	Zero stage 2
Epoch (steps)	3 (115)	3 (115)
Learning rate scheduler	Cosine	Cosine
Warmup ratio (% of steps)	0.03	0.03
Peak learning rate	0.0001	0.0001
Optimizer	AdamW	AdamW
Weight decay	0.01	0.01
Maximum sequence length	2048	2048
Data type	float32	float32
LoRA^b^ rank	64	64
LoRA α	128	128
LoRA weights	Query, key, value, output, MLP^c^	Query, key, value, output, MLP
LoRA dropout	0.05	0.05

^a^GPU: graphics processing unit.

^b^LoRA: Low-Rank Adaptation.

^c^MLP: multilayer perceptron.

**Prompting template for GPT-4 evaluation.**

You are a super-intelligent AI assistant.

question: {question}

context: {Title: title, Date: date, Country: country, Source: source, Contents: article}

Considering the context provided, determine which answer is the best answer for the given question and context. The answer should point out the what the question requires. You must choose only one among options below.

* Answer A

* Answer B

* Answer C

* Answer N

Initially, GPT-4 evaluation was used to assess the performance of AceGPT-7B-Chat, InfectA-Chat (based on AceGPT-7B), and InfectA-Chat (based on AceGPT-7B-Chat). Subsequently, the model demonstrating superior performance in the first phase underwent evaluation against Arabic LLMs, including Jais-13B-Chat and AceGPT-13B-Chat, in the second phase. Finally, building upon the results of the second phase, the selected model was evaluated against state-of-the-art LLMs, such as GPT-3.5, GPT-4, and Gemini, in the last phase. Furthermore, to assess the generalizability of the superior model from the InfectA-Chat suite (comprising AceGPT-7B and AceGPT-7B-Chat), we extended our evaluation beyond the infectious disease domain. We used a broad dataset built for tasks that required common-sense reasoning and Q&A skills. This cross-domain testing enabled us to assess the model’s capacity to use its learned information and reasoning skills in a broader environment, offering vital insights into its overall robustness and adaptability. This enabled a full evaluation of model performances.

### Experimental Result

#### Overview

**Figure 5:**
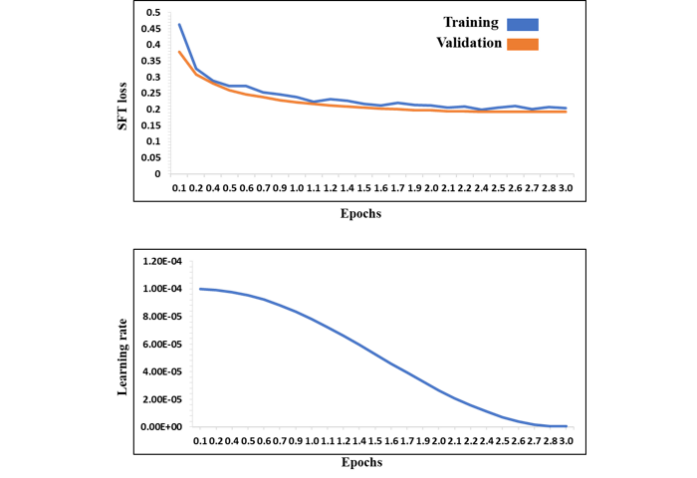
Training progress of AceGPT-7B–based InfectA-Chat (training loss, validation loss, and learning rate). SFT: supervised fine-tuning.

**Figure 6:**
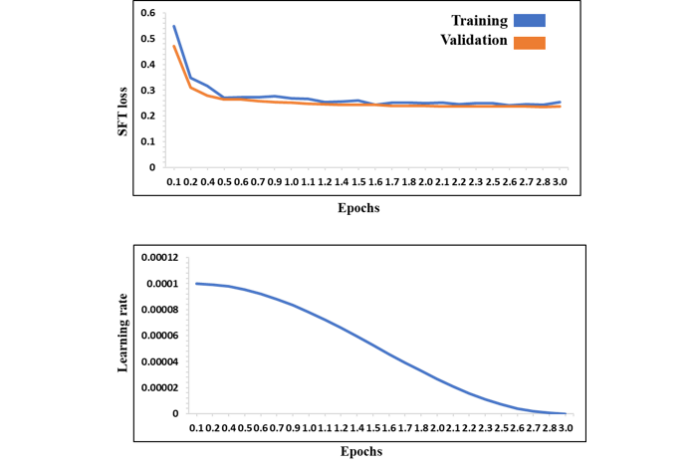
Training progress of AceGPT-7B-Chat–based InfectA-Chat (training loss, validation loss, and learning rate). SFT: supervised fine-tuning.

We present the training progress for the InfectA-Chat models (based on AceGPT-7B and AceGPT-7B-Chat), which are visualized in Figures 5 and 6, respectively. Both figures depict a learning rate starting at 0.0001 and gradually decreasing due to the learning rate scheduler used for optimizing convergence. The loss curves in both figures reveal a steady decrease, demonstrating both models effectively learned from the training data and generalized well to unseen data.

#### Performance Comparison of InfectA-Chat in the Infectious Disease Domain

**Figure 7:**
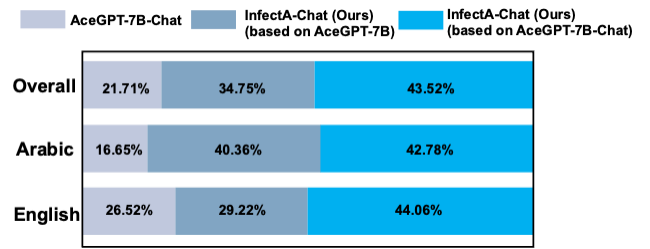
Qualitative performance comparison for AceGPT-7B-Chat, AceGPT-7B–based InfectA-Chat (ours), and AceGPT-7B-Chat–based InfectA-Chat (ours) by GPT-4 evaluation.

**Figure 8:**
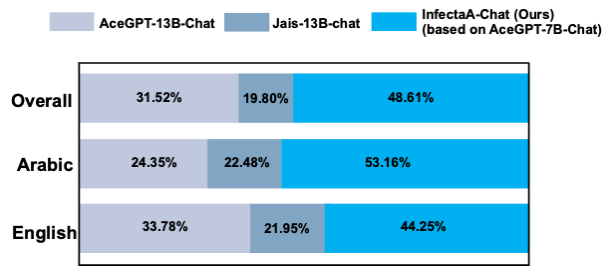
Qualitative performance comparison for AceGPT-13B-Chat, Jais-13B-Chat, and AceGPT-7B-Chat–based InfectA-Chat (ours) by GPT-4 evaluation.

**Figure 9:**
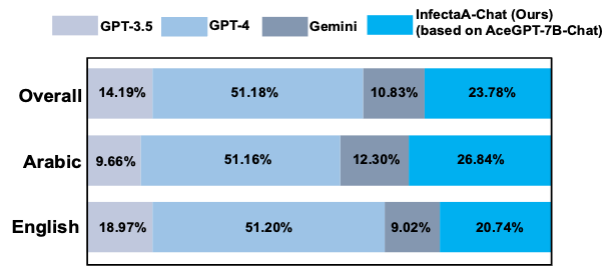
Qualitative performance comparison for InfectA-Chat (ours), GPT-4, GPT-3.5, and Gemini.

The results of qualitative performance comparisons were conducted across 3 phases using GPT-4 (Figures 7-9). The decision to exclude base models among Arabic LLMs during GPT-4 evaluation was motivated by the number of errors observed in the base models. During the GPT-4 evaluation on AceGPT-7B, 102 errors occurred. In terms of AceGPT-13B and Jais-13B evaluation process, 41 and 669 errors occurred, respectively. Due to the generation of improper answers, GPT-4 could not process during the evaluation. The inclusion of the AceGPT-7B, AceGPT-13B, and Jais-13B models in the comparison study may have a negative impact on the performance analysis’s accuracy. Therefore, these 3 base models were omitted from the GPT-4 evaluation to enable a more accurate assessment of model performance. The performance results represent the average accuracy of the 5-step GPT-4 evaluation, which is depicted in Table 2.

**Table 2:** Qualitative performance analysis of 5-step GPT-4 evaluation process.

Evaluation phases and models		Accuracy					
		Step 1	Step 2	Step 3	Step 4	Step 5	Mean (SD)
**Phase 1 (%)**							
	InfectA-Chat (based on AceGPT-7B-Chat)	43.86	43.93	42.77	43.61	43.46	43.52 (0.46)
	InfectA-Chat (based on AceGPT-7B)	34.99	34.22	35.66	34.51	34.40	34.75 (0.58)
	AceGPT-7B-Chat	21.15	21.85	21.57	21.88	22.12	21.71 (0.37)
**Phase 2 (%)**							
	InfectA-Chat (based on AceGPT-7B-Chat)	47.44	51.27	50.37	46.53	47.76	48.61 (0.20)
	AceGPT-13B-Chat	32.35	31.37	31.22	32.82	29.86	31.52 (0.11)
	Jais-13B-Chat	20.21	17.36	18.41	20.65	22.38	19.8 (0.19)
**Phase 3 (%)**							
	InfectA-Chat (based on AceGPT-7B-Chat)	24.08	23.21	23.93	24.33	23.36	23.78 (0.47)
	Gemini	10.43	11.12	10.76	10.52	11.33	10.83 (0.38)
	GPT-3.5-Turbo	14	13.54	14.56	14.18	14.70	14.19 (0.46)
	GPT-4	51.49	52.13	50.76	50.97	50.61	51.18 (0.62)

In the initial phase, InfectA-Chat (based on AceGPT-7B-Chat) demonstrated superior performance compared to AceGPT-7B-Chat and InfectA-Chat (based on AceGPT-7B), achieving an accuracy of 43.52% on the overall instruction-following dataset (Figure 7). Notably, for English and Arabic instruction–following data, InfectA-Chat (based on AceGPT-7B-Chat) surpassed other models, achieving performance rates of 44.06% on 614 of 1393 English test cases and 42.78% on 589 of 1377 Arabic test cases, respectively. In addition, the performance gap between AceGPT-7B-Chat and InfectA-Chat (based on AceGPT-7B-Chat) can also be observed in their answers to Arabic and English queries (Multimedia Appendix 1).

In comparison to other Arabic LLMs such as AceGPT-13B-Chat and Jais-13B-Chat, InfectA-Chat (based on AceGPT-7B-Chat) showed better performance, achieving an overall performance rate of 48.61% on 1346 of 2770 overall test cases in the instruction-following dataset (Figure 8). Particularly, its performance in English and Arabic instruction–following data, where InfectA-Chat (based on AceGPT-7B-Chat) surpassed other models, achieving rates of 44.25% (616/1393 English test cases) and 53.16% (732/1377 Arabic test cases), respectively. These results prove the capability of InfectA-Chat (based on AceGPT-7B-Chat) not only to outperform similar-scale Arabic LLMs but also to compete favorably against larger models with up to 13 billion parameters.

As the final step, in addition to comparing with open-source Arabic LLMs, the InfectA-Chat (based on AceGPT-7B-Chat) model was evaluated against state-of-the-art closed-source models, including GPT-3.5, GPT-4, and Gemini (Figure 9). These comparisons revealed that the InfectA-Chat (based on AceGPT-7B-Chat) model substantially outperformed GPT-3.5 and Gemini, achieving a performance rate of 23.78% on 656 of 2770 test cases while competing closely with the GPT-4 models, which attained a performance rate of 51.18% (1417/2770 test cases) against other models. This highlights the competitive edge of InfectA-Chat (based on AceGPT-7B-Chat) against top-tier closed-source models in the field of infectious diseases.

As demonstrated, we used GPT-4 as an evaluation metric. However, since GPT-4 can show variability during generating answers based on our prompts, we used SD to ensure the trustworthiness of this evaluation method. To do that, we applied GPT-4 evaluation 5 times to our 3-step evaluation process and we compared each model’s accuracy in each evaluation step and observed their SD. The further details of this analysis are depicted in Table 2.

As we can see in the SD results from Table 2, the SD of each model in each phase is quite low. This proves the consistency of our GPT-4 evaluation method that reflects our comparison model’s performance.

#### Performance Comparison of InfectA-Chat in the General Domain

To evaluate the generalizability of the best-performing InfectA-Chat model (based on AceGPT-7B-Chat), we conducted cross-domain testing on the Arabic Massive Multitask Language Understanding (MMLU) [54] benchmark (Table 3). This dataset assesses common-sense reasoning and Q&A capabilities, crucial for tasks beyond the infectious disease domain. The Arabic MMLU dataset comprises 40 tasks and 14,575 multiple-choice questions in Modern Standard Arabic, meticulously constructed in collaboration with native Arabic speakers. This dataset covers a wide range of subjects, including science, technology, engineering, and mathematics (STEM); social sciences; humanities; and others, ensuring its relevance as a source of common-sense knowledge. The questions are designed to assess a spectrum of knowledge levels, from primary education to university-level understanding. Because the dataset is specifically tailored to evaluate models’ real-world knowledge, we chose to use the Arabic MMLU dataset to assess our model’s performance on practical data. We evaluated InfectA-Chat and other models’ performance in a multiple-choice Q&A task in 4 different domains, such as STEM, humanities, social sciences, and others, as reflected in Table 3. In the STEM domain, InfectA-Chat achieved a performance score of 40.84% on 1315 of 3220 STEM data, outperforming the LLaMA models (LLaMA-7B and LLaMA-13B) as well as the Jais base models (Jais-13B and Jais-30B) and Bloomz. Its performance was on par with that of the more advanced AceGPT chat models (AceGPT-7B-Chat and AceGPT-13B-Chat), Jais chat models (Jais-13B-Chat and Jais-30B-v3-Chat), and GPT-3.5-Turbo, indicating its strong capabilities in technical subjects. In the humanities domain, InfectA-Chat scored 46.94% on 1715 of 3655 humanities data, demonstrating higher accuracy than LLaMA models, Bloomz, Jais base models, AceGPT base models, and GPT-3.5-Turbo. Its results closely approach those of the AceGPT chat models (48.54% for AceGPT-7B-Chat and 50.91% for AceGPT-13B-Chat, with the following 1774 and 1861 data points out of 3655) as well as the Jais chat models. In the social sciences and other general domains, InfectA-Chat achieved scores of 45.58% (1613/3540 social sciences-related data) and 49.48% (1751/3540 general domain data), respectively, surpassing LLaMA models, AceGPT base models, Bloomz, and Jais base models. Instruction tuning is one of the primary factors limiting the generalizability of InfectA-Chat. Despite this, our model, which is specifically focused on the infectious diseases domain, demonstrates performance comparable to that of the AceGPT and Jais chat models, highlighting its adaptability and strong performance across a wide range of disciplines. On average, across 4 domains, InfectA-Chat achieved a strong overall score of 45.71% on 6662 of 14,575 general domain data, positioning it competitively among large multilingual models such as Bloomz (34.53% of 14,575 data), LLaMA-7B (30.78% of 14,575 data), and LLaMA-13B (37.27% of 14,575 data). Notably, InfectA-Chat outperformed leading Arabic pretrained models, such as AceGPT-13B and the Jais-series base models (Jais-13B-base and Jais-30B-base), with a performance gap ranging from 2.99% to 12.28% of 14,575 data. This suggests that InfectA-Chat is especially effective at handling common-sense multiple-choice Q&A tasks compared to other prominent Arabic models. Its performance also closely approaches that of instruction-tuned models such as AceGPT-7B-Chat (47.58% performance rate on 6934/14,575 data) and AceGPT-13B-Chat (50.81% performance rate on 7405/14,575 data) while remaining competitive against advanced Jais-series chat models (Jais-13B-Chat and Jais-30B-v3-Chat), which scored between 55.11% and 61.14% of 14,575 data, respectively. This underscores InfectA-Chat’s significant performance not only in the domain of infectious diseases but also in real-world common-sense data, outperforming both English and Arabic base models while maintaining competitive results with a small performance gap with Arabic chat models.

**Table 3:** The results of testing Arabic large language models on the Arabic Massive Multitask Language Understanding benchmark dataset (accuracy).

Model	Mean (%)	STEM^a^ (%)	Humanities (%)	Social sciences (%)	Others (%)
Bloomz	34.53	33.35	29.29	37.58	34.53
LLaMA2-7B	30.78	30.30	29.33	27.46	30.78
LLaMA2-13B	37.27	32.94	32.30	33.42	37.27
Jais-13B-based	33.43	30.51	31.25	33.74	33.43
Jais-30B-based	39.60	32.67	30.67	42.13	39.60
AceGPT-7B-based	34.42	29.73	30.95	33.45	34.42
AceGPT-13B-based	42.72	36.60	38.74	43.76	42.72
AceGPT-7B-Chat	47.58	42.41	48.54	47.03	52.33
AceGPT-13B-Chat	50.81	47.10	50.91	50.97	54.27
Jais-13b-Chat	55.11	49.98	56.73	54.05	59.66
Jais-30b-v3-Chat	61.14	54.59	63.45	59.10	67.43
GPT-3.5-Turbo	49.07	43.38	44.12	55.57	53.21
InfectA-Chat	45.71	40.84	46.94	45.58	49.48

^a^STEM: science, technology, engineering, and mathematics.

#### Advanced InfectA-Chat Performance With RAG Pipeline

**Figure 10:**
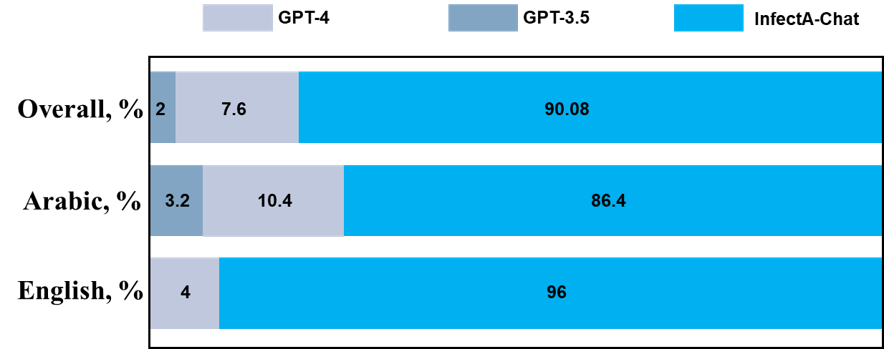
Qualitative performance results for InfectA-Chat on the recent dataset (March 20, 2024, to June 20, 2024) by GPT-4 evaluation.

Traditionally, recurrent fine-tuning of models with additional data incurs significant time and computational costs, making it an inefficient strategy. To address this issue, the implementation of the RAG pipeline is critical. In our study, we validated the necessity by applying the RAG pipeline to InfectA-Chat alongside cutting-edge models such as GPT-3.5 and GPT-4, using a set of 50 recent instruction-following data. The results illustrated the efficacy of the RAG pipeline when incorporating recent data (Figure 10). The results in Figure 10 were obtained by averaging the inference accuracy of each model across 5 runs in English and Arabic data to prove the consistency of model performances. Across all datasets, InfectA-Chat exhibited a remarkable performance of 90.08% on 45 of 50 RAG test data, with an SD of 1.7%. Particularly in the Arabic dataset, InfectA-Chat demonstrated superior performance at 86.4% (43/50 RAG test data), and in the English dataset, it excels at 96% on 48 of 50 RAG test data, outperforming both GPT-3.5 and GPT-4 models, which have SD of 0.8% (4/50 RAG test data) and 0.16% (8/50 RAG test data), respectively. These findings highlight the capacity of RAG to obviate the need for repetitive instruction tuning by integrating the model with up-to-date information, particularly crucial in domains such as public health and infectious diseases.

**Figure 11:**
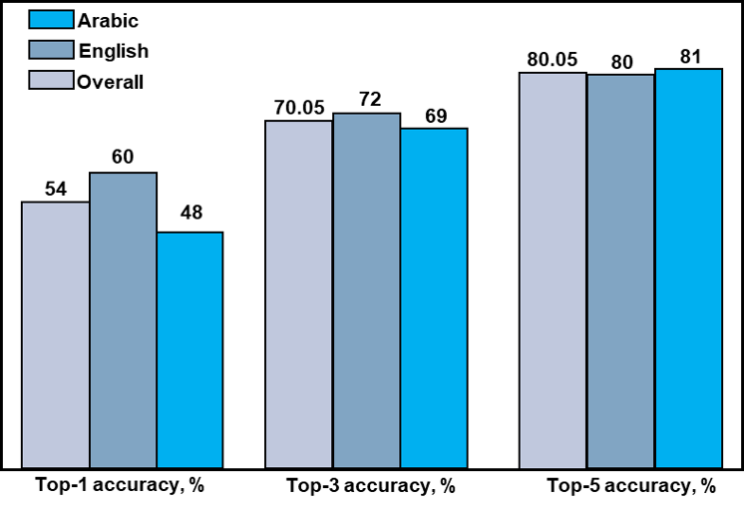
Accuracy of retriever based on top-k parameter in Retrieval-Augmented Generation pipeline.

Following the results of the RAG pipeline with the up-to-date dataset, to delve deeper into the influence of information retrieval on factual language models like InfectA-Chat, we investigated the impact of the top-k parameter within the RAG pipeline. This parameter controls the number of top-ranked documents passed from the retrieval stage to the generation stage. Our experiments with RAG using top-1, top-3, and top-5 settings demonstrated a positive relationship between increasing the top-k value and the accuracy of retrieval (Figure 11).

This implied that exposing InfectA-Chat to a greater range of potentially relevant information during retrieval can considerably improve its factual language modeling capabilities. As observed, this translated to a substantial performance improvement for InfectA-Chat, with an accuracy of 80.05% on 40 of 50 RAG test data, achieved using the top-5 parameter compared to the top-1.

## Discussion

In conclusion, the critical need for real-time disease monitoring, particularly in regions like the Middle East, has exposed a gap in accessible, multilingual resources. Regional public health agencies, such as the US Centers for Disease Control and Prevention [55] and the Chinese Center for Disease Control and Prevention [56], play an important role in protecting public health by conducting research and offering disease surveillance and prevention programs in their regions. In addition, there has been a rise in innovative platforms providing daily alerts, such as the CIDRAP [45] and the Program for Monitoring Emerging Diseases [57]. Apart from these platforms, with the rise of LLMs, LLMs started to play a role in delivering information in various domains, including infectious disease tracking. Given our focus on Arabic-speaking countries within the MENA region, there remain a limited number of Arabic language models specifically tailored for the medical and public health domains. While the development of medical LLMs is expanding globally, only a few models, such as BiMediX [38] and Apollo [39], have been created with a focus on Arabic language capabilities. Although these models are valuable for supporting societal and medical initiatives, they still face limitations, especially in monitoring infectious diseases, due to the limited availability of infectious disease surveillance data for training. InfectA-Chat addresses this challenge by delivering up-to-date information on infectious diseases in both Arabic and English. Moreover, the exceptional performance of InfectA-Chat in handling instruction-following data in Q&A tasks across languages sets a new standard in the field of infectious disease monitoring. By leveraging the power of LLMs, InfectA-Chat positions itself as a pioneer for bridging the information gap and promoting public health awareness compared to other existing LLMs. However, even though InfectA-Chat has significant performance, it still shows limitations itself.

First, while InfectA-Chat demonstrates significant performance in Arabic and English understanding, it may still face challenges due to limited computing resources and data availability. The limitation in the amount of data available in English and Arabic in the field of infectious diseases create a barrier to the model’s performance. In addition, because the training of LLMs requires more computational resources, such as GPUs, this would make the development process faster and more efficient. Due to our limited computational resources, the process becomes slower, restricting us to using smaller model sizes. As the accessibility of computational resources and instruction-following data continue to grow, InfectA-Chat can achieve greater performance.

Second, in some scenarios, the model may generate inconsistent answers based on the given questions, such as incorrect information about specific diseases, known as the hallucination problem. Although this issue was mitigated by implementing a RAG pipeline, InfectA-Chat may still exhibit a small likelihood of hallucination. Applying RAG with a larger volume of data could further reduce the hallucination rate to nearly 0.

Future work efforts should focus on mitigating these limitations to unlock the potential of InfectA-Chat. With larger datasets and more computational resources, the performance of InfectA-Chat can be increased. In addition, we will focus on how to improve the performance of our model in the general domain by mitigating the disadvantages of instruction tuning. We aim to establish InfectA-Chat as a truly transformative tool in the fight against infectious diseases by enhancing our target regions and obtaining massive data in different languages.

## Appendix

Multimedia Appendix 1 Additional examples of generated answers based on questions in Arabic and English. Arabic examples are provided with their English translations.

## Abbreviations

BERT: Bidirectional Encoder Representations from Transformers
BiMediX: Bilingual Medical Mixture of Experts Large Language Model
BioBERT: BERT for Biomedical Text Mining
CIDRAP: Center for Infectious Disease Research and Policy
ECDC: European Centre for Disease Prevention and Control
GPU: graphics process unit
LLM: large language model
LoRA: low-rank adaptation
MERS-CoV: Middle East respiratory syndrome coronavirus
MENA: Middle East and North Africa
MIMIC-III: Medical Information Mart for Intensive Care III
MMLU: Massive Multitask Language Understanding
NLP: natural language processing
PaLM: Pathways Language Model
PEFT: parameter-efficient fine-tuning
Q&A: question and answer
RAG: retrieval-augmented generation
STEM: science, technology, engineering, and mathematics
WHO: World Health Organization
