# Supplementary material for: InfectA-Chat, an Arabic Large Language Model for Infectious Diseases: Comparative Analysis
Source: JMIR Med Inform. 2025 Feb 10;13:e63881. doi: 10.2196/63881 (PMC11851044; doi:10.2196/63881)
Supplement: Multimedia Appendix 1 [file medinform_v13i1e63881_app1.pdf]

Generated Answers for Infectious Disease-related Question in Arabic

<table>
<tr><td colspan="2">Prompt: ما هي تفاصيل التعرض التي قدمتها منظمة الصحة العالمية فيما يتعلق بالرجل القطري البالغ من العمر 73 عامًا والمصاب بفيروس كورونا؟
(What were the exposure details provided by the WHO regarding the 73-year-old Qatari man with MERS?)</td></tr>
<tr><td>AceGPT-7B-Chat</td><td>آسف، لا أستطيع تزويدك بأي معلومات حيث لا يوجد سؤال محدد. يرجى تزويدي بسؤال واضح وموجز حتى أتمكن من مساعدتك وفقًا لذلك.

(I am sorry, I cannot provide you with any information as there is no specific question asked. Please provide me with a clear and concise question so that I can assist you accordingly.)</td></tr>
<tr><td>InfectA-Chat</td><td>وأوضحت منظمة الصحة العالمية أن الرجل القطري البالغ من العمر 73 عامًا والمصاب بفيروس كورونا تعرض للعدوى من خلال اتصال أفراد أسرته بالإبل واستهلاك حليب الإبل الخام.

(The WHO detailed that the 73-year-old Qatari man with MERS was exposed through his family members having contact with camels and consuming raw camel milk.)</td></tr>
<tr><td colspan="2">Prompt: ما هو تاريخ حالة إصابة جديدة بفيروس كورونا المسبب لمتلازمة الشرق الأوسط؟
(What is the history of a new case of MERS-CoV?)</td></tr>
<tr><td>AceGPT-7B-Chat</td><td>أعتذر على التأخير، ولكن يبدو أن المعلومات المطلوبة تتعلق بتاريخ حديث للغاية وليس لدي تفاصيل حول "تاريخ حالة إصابة جديدة بفيروس كورونا المسبب لمتلازمة الشرق الأوسط" في 20 ديسمبر 2018. ومع ذلك، يمكنني تقديم معلومات عامة حول فيروس كورونا المسبب لمتلازمة الشرق الأوسط (MERS-CoV). MERS هو فيروس تم اكتشافه لأول مرة في عام 2012 في المملكة العربية السعودية وقد أثار مخاوف عالمية بسبب عدد الوفيات الناجمة عنه. الأعراض الأكثر شيوعًا لـ COVID-19، المرض الناجم عن الفيروس، تشمل الحمى، السعال، وصعوبة التنفس. بعض الأشخاص قد يعانون من أعراض أخرى مثل التعب، وآلام الجسم، والتهاب العين، والتهاب الحلق، والتنفس الصعب. الفيروس ينتقل عادة من شخص لآخر عن طريق القطيرات التنفسية عندما يسعل أو يعطس الشخص المصاب. لا يوجد حتى الآن علاج محدد لـ COVID-19، ولكن العديد من الأدوية والتطعيمات قيد التجربة. العلاج الرئيسي يركز على تخفيف الأعراض ودعم وظائف الجسم الحيوية. الوقاية من الفيروس تتضمن تجنب التواصل القريب مع الأشخاص المصابين بأعراض خطيرة واتباع الإرشادات الصحية العامة، مثل غسل اليدين بانتظام وتجنب لمس الوجه. أتمنى أن يكون هذا الرد مفيدًا ويرجى مراجعة المصادر الرسمية للحصول على أحدث المعلومات حول MERS-CoV وCOVID-19.

(I apologize for the delay, but it appears that the information requested is for a very recent date and I do not have details about the "date of a new MERS case" on December 20, 2018. However, I can provide general information about MERS - CoV). MERS is a virus that was first discovered in 2012 in Saudi Arabia and has raised global concerns due to the number of deaths caused by it. The most common symptoms of COVID-19, the disease caused by the virus, include fever, cough, and difficulty breathing. Some people may experience</td></tr>
</table>

	other symptoms such as fatigue, body aches, eye inflammation, sore throat, and difficult breathing. The virus is usually transmitted from person to person through respiratory droplets when an infected person coughs or sneezes. There is still no specific treatment for COVID-19, but several drugs and vaccinations are under trial. The main treatment focuses on relieving symptoms and supporting vital body functions. Preventing the virus includes avoiding close contact with people with serious symptoms and following general health guidelines, such as washing hands regularly and avoiding touching the face. I hope you found this response helpful and please check official sources for the latest information about MERS-CoV and COVID-19.)
**InfectA-Chat**	سُجلت حالة إصابة جديدة بفيروس كورونا المسبب لمتلازمة الشرق الأوسط التنفسية للأسبوع الوبائي 51.<br>***(One new case of infection with the Middle East respiratory syndrome coronavirus was recorded for epidemiological week 51.)***
**Prompt:** كيف بقيت الوفيات نتيجة الإنفلونزا والالتهاب الرئوي؟<br>***(How did deaths remain as a result of influenza and pneumonia?)***	
**AceGPT-7B-Chat**	أعتذر على التأخير في الرد. بناءً على التحديث الأخير من مراكز السيطرة والوقاية من الأمراض، تظل نسبة الإصابة بالإنفلونزا في الولايات المتحدة منخفضة في الوقت الحالي. ومع ذلك، يُشدد على ضرورة التطعيم قبل زيادة الإصابات. على المستوى العالمي، تظل الدول مثل العراق والبحرين وقطر تبلغ عن زيادة في نشاط الإنفلونزا، بعضها بسبب فيروس H1N1. ومع ذلك، يظل مستوى الإنفلونزا عامة منخفضًا أو في تراجع باستثناء بعض الدول التي تبلغ عن زيادة نشاط الإنفلونزا من فيروس H1N1. وفقًا للمنظمة العالمية للصحة، نشاط الإنفلونزا في نصف الكرة الشمالي لا يزال عند مستويات بين مواسمية، مع كشف عشوائي عن الإنفلونزا. أتمنى أن يكون هذا الرد مفيدًا لك. إذا كان لديك أي أسئلة أخرى، فلا تتردد في طرحها.<br><br>***(I apologize for the delay in responding. Based on a recent update from the Centers for Disease Control and Prevention, the incidence of influenza in the United States currently remains low. However, he stresses the necessity of vaccination before infections increase. Globally, countries such as Iraq, Bahrain and Qatar continue to report an increase in influenza activity, some due to the H1N1 virus. However, the overall level of influenza remains low or declining with the exception of some countries reporting increased influenza activity from the H1N1 virus. According to the World Health Organization, influenza activity in the Northern Hemisphere remains at inter-seasonal levels, with random detection of influenza. I hope this response is useful to you. If you have any further questions, feel free to ask.)***
**InfectA-Chat**	بقيت الوفيات نتيجة الإنفلونزا والالتهاب الرئوي بعيدة تمامًا عن القاعدة الموسمية.<br>***(Five deaths from influenza and pneumonia remained well outside the seasonal norm.)***

**Prompt:** كم عدد الحالات التي أعلنت في ولاية آنهوي والتي شملت حالة تم الإبلاغ عنها بالفعل؟<br>***(How many cases announced in Anhui Province included an already reported case?)***	
**AceGPT-7B-Chat**	أعتذر على الخطأ، ولكن يبدو أن المعلومات المقدمة في الطلب غير صحيحة. لا يوجد معلومات حول حالات MERS-CoV في ولاية آنهوي في المصدر المقدم. بالإضافة إلى ذلك، لا يوجد معلومات حول حالات فيروس H7N9 في شنغهاي في المصدر المقدم. من الأفضل تقديم معلومات صحيحة ودقيقة لتجنب نشر معلومات خاطئة. أعتذر عن الإرباك وأتمنى لك يومًا سعيدًا!<br><br>***(I apologize for the error, but it appears that the information provided in the application is incorrect. There is no information about MERS-CoV cases in Anhui Province in the provided source. In addition, there is no information about H7N9 cases in Shanghai in the provided source. It is best to provide true and accurate information to avoid spreading false information. I apologize for the confusion and have a nice day!)***
**InfectA-Chat**	خمس حالات، منها واحدة تم الإبلاغ عنها بالفعل.<br>***(Five cases, one of which has already been reported.)***

Generated Answers for Infectious Disease-related Question in English

<table>
<tr><td colspan="2">Prompt: What mutation concerns were raised regarding H9N2 avian influenza viruses found in poultry in Bangladesh?</td></tr>
<tr><td>AceGPT-7B-Chat</td><td>Thank you for your inquiry. I am unable to provide information related to specific news articles or reports without access to the full text of those documents. However, based on the information provided in your question, it appears that the article discusses several different topics, including the implementation of a new strategy for improving hand hygiene among healthcare workers, the increase in West Nile virus cases in the United States, the discovery of mutations in H9N2 avian influenza viruses in Bangladesh, and the continuing investigation into the 2012 fungal meningitis outbreak. If you have any additional questions or need assistance with anything else, please let me know.</td></tr>
<tr><td>InfectA-Chat</td><td>Mutations in H9N2 avian influenza viruses found in poultry in Bangladesh suggested they might be adapting to mammals, potentially including humans.</td></tr>
<tr><td colspan="2">Prompt: What specific recommendation was given to travelers planning to visit Israel to protect against measles?</td></tr>
<tr><td>AceGPT-7B-Chat</td><td>I am sorry but I cannot complete this task because it requires me to access external sources and provide information that may be subject to change without notice. As a language model, my responses are based solely on patterns found in the text provided to me and do not reflect real-time events or updates. If you need assistance with a different topic, please let me know.</td></tr>
<tr><td>InfectA-Chat</td><td>The recommendation was to protect oneself against measles by getting vaccinated at least two weeks in advance of the trip to Israel.</td></tr>
<tr><td colspan="2">Prompt: When did the 16-year-old girl in Egypt succumb to the H5N1 avian influenza infection?</td></tr>
<tr><td>AceGPT-7B-Chat</td><td>I am sorry but I cannot provide you with information regarding the date or time of the 16-year-old girl's death as it is not specified in the provided article. However, based on the information given in the article, the girl passed away due to complications caused by the H5N1 avian influenza infection. It is important to note that this is a serious disease and can be fatal if left untreated. If you are concerned about your own health or the health of someone else, please consult with a medical professional immediately.</td></tr>
<tr><td>InfectA-Chat</td><td>The 16-year-old girl in Egypt succumbed to the H5N1 avian influenza infection on December 16, 2008.</td></tr>
<tr><td colspan="2">Prompt: What is the current total number of MERS-CoV cases and deaths reported by the Saudi Ministry of Health?</td></tr>
<tr><td>AceGPT-7B-Chat</td><td>According to the latest updates from the Saudi Ministry of Health, there are currently three additional cases of MERS-CoV in Saudi Arabia, two of which are severe. Additionally, there has been one reported death due to MERS-CoV in the Eastern region. As for Qatar, they have reported their seventh case of MERS-CoV, which is a non-Qatari individual who is asymptomatic. However, it is important to note that these numbers may change as further investigations are conducted.</td></tr>
<tr><td>InfectA-Chat</td><td>The Saudi Ministry of Health has reported a total of 124 MERS-CoV cases with 52 deaths.</td></tr>
</table>
